# Determinants of Deadwood-Inhabiting Fungal Communities in Temperate Forests: Molecular Evidence From a Large Scale Deadwood Decomposition Experiment

**DOI:** 10.3389/fmicb.2018.02120

**Published:** 2018-09-20

**Authors:** Witoon Purahong, Tesfaye Wubet, Guillaume Lentendu, Björn Hoppe, Katalee Jariyavidyanont, Tobias Arnstadt, Kristin Baber, Peter Otto, Harald Kellner, Martin Hofrichter, Jürgen Bauhus, Wolfgang W. Weisser, Dirk Krüger, Ernst-Detlef Schulze, Tiemo Kahl, François Buscot

**Affiliations:** ^1^Department of Soil Ecology, Helmholtz Centre for Environmental Research – UFZ, Halle, Germany; ^2^German Centre for Integrative Biodiversity Research (iDiv), Leipzig, Germany; ^3^Department of Ecology, Technical University of Kaiserslautern, Kaiserslautern, Germany; ^4^Institute for National and International Plant Health, Julius Kühn-Institute, Braunschweig, Germany; ^5^Department of Biology and Environmental Sciences, International Institute Zittau, Technische Universität Dresden, Zittau, Germany; ^6^Department of Systematic Botany and Functional Biodiversity, Institute of Biology, University of Leipzig, Leipzig, Germany; ^7^Department of Molecular Evolution and Plant Systematics, Institute of Biology, University of Leipzig, Leipzig, Germany; ^8^Chair of Silviculture, Faculty of Environment and Natural Resources, University of Freiburg, Freiburg im Breisgau, Germany; ^9^Terrestrial Ecology Research Group, Department of Ecology and Ecosystem Management, School of Life Sciences Weihenstephan, Technical University of Munich, Freising, Germany; ^10^Max Planck Institute for Biogeochemistry, Jena, Germany; ^11^Biosphere Reserve Vessertal-Thuringian Forest, Schmiedefeld am Rennsteig, Germany

**Keywords:** next generation sequencing, microbial ecology, BELongDead, wood-physicochemical properties, fungal richness, fungal community composition

## Abstract

Despite the important role of wood-inhabiting fungi (WIF) in deadwood decomposition, our knowledge of the factors shaping the dynamics of their species richness and community composition is scarce. This is due to limitations regarding the resolution of classical methods used for characterizing WIF communities and to a lack of well-replicated long-term experiments with sufficient numbers of tree species. Here, we used a large scale experiment with logs of 11 tree species at an early stage of decomposition, distributed across three regions of Germany, to identify the factors shaping WIF community composition and Operational Taxonomic Unit (OTU) richness using next generation sequencing. We found that tree species identity was the most significant factor, corresponding to (*P* < 0.001) and explaining 10% (representing 48% of the explainable variance) of the overall WIF community composition. The next important group of variables were wood-physicochemical properties, of which wood pH was the only factor that consistently corresponded to WIF community composition. For overall WIF richness patterns, we found that approximately 20% of the total variance was explained by wood N content, location, tree species identity and wood density. It is noteworthy that the importance of determinants of WIF community composition and richness appeared to depend greatly on tree species group (broadleaved *vs*. coniferous) and it differed between the fungal phyla Ascomycota and Basidiomycota.

## Introduction

Deadwood is an important structural component of forest ecosystems and an essential part of the nutrient cycle (Rajala et al., 2012; Stokland et al., 2012; Purahong et al., 2017; Herrmann and Bauhus, 2018). Deadwood can also be considered to act as a hot spot for biodiversity as millions of species (including bacteria, fungi, plants and animals) are considered to be associated with deadwood (Stokland et al., 2012). Deadwood is a complex substrate comprising a variety of components, ranging from simple molecules (e.g., organic acids, sugars) to complex biopolymers such as cellulose, heteropolysaccharides and lignin, that are difficult to decompose (Floudas et al., 2012). Wood-inhabiting fungi (WIF), especially saprotrophs (i.e., white-rot, brown-rot and soft-rot fungi) play important roles in the decomposition and mineralisation processes of downed deadwood, and they recycle nutrients in various forms to render them accessible to other organisms (Wei and Dai, 2004). Apart from saprotrophs, WIF also include other functional groups inhabiting deadwood such as, ectomycorrhizal, lichenised, mycoparasitic and plant pathogenic fungi (Purahong et al., 2017, 2018a). Due to their importance for biodiversity and ecosystem functioning, they have been increasingly studied in recent years (Seibold et al., 2015; Bauhus et al., 2018).

Traditionally, studies of WIF mainly employ fruiting body surveys and various isolation methods (Bader et al., 1995; Allmér et al., 2006). The results from such approaches are useful but may not cover the majority of fungi inhabiting deadwood (Kubartová et al., 2012). Biases and potential pitfalls from using fruiting body surveys and culturing methods have been discussed extensively (Kubartová et al., 2012; Hoppe et al., 2016). As an alternative, culture-independent molecular approaches have been applied recently to characterize the WIF community across different biomes and continents to increase our understanding of WIF diversity and distribution (Hiscox et al., 2015; Ottosson et al., 2015; Hoppe et al., 2016; Purahong et al., 2017).

Fungal diversity and community composition change with wood-physicochemical properties during the course of decomposition (Rajala et al., 2012; Baldrian et al., 2016). Both factors at the level of the individual piece of deadwood, for example wood-physicochemical properties (especially pH and macronutrients), and tree species identity, as well as plot-related factors (i.e., forest structure and composition, current and past forest management, slope and elevation) influence WIF community composition (Rajala et al., 2011, 2012; Purahong et al., 2014a,b, 2016a, 2017; Baldrian et al., 2016; Hoppe et al., 2016). Among the significant factors, wood-physicochemical properties and tree species identity have been found to be the major influences on WIF communities across different experiments and locations (Baldrian et al., 2016; Purahong et al., 2016a, 2017). However, no experiment to date has quantified the respective contributions of such factors on WIF community composition (as well as species richness) and all evidence we have obtained to date, especially from the more reliable assessment of the total fungal community using molecular, culture-independent techniques originated mostly from one or a few tree species, which limits the robustness of the conclusions (Purahong et al., 2017). Comparisons between two tree species such as between *Fagus sylvatica* and *Picea abies*, or *Schima superba* and *Pinus massoniana* revealed that differences in fungal communities between different hosts resulted from their highly distinct wood-physicochemical properties (Hoppe et al., 2016; Purahong et al., 2017). It is, however, still unclear how WIF communities vary between a larger number of tree species that represent more gradual differences in wood-physicochemical properties. Furthermore, from an experiment in Central Europe, we learned that the wood substrate used for field experiments should be comparable, especially in terms of the time since death of the analyzed trees, as it is otherwise very difficult to distinguish the effects of wood-physicochemical properties from those of the decay stage (corresponding to time since death) (Allmér et al., 2006). Thus, experiments combining high diversities of deadwood species and starting with homogenous log material are still needed.

Recently, we have established one of the largest ever deadwood decomposition experiments, comprising 13 tree species from Central European forests situated in 27 plots covering a diverse range of forest conditions over three regions across Germany. Of the 13 tree species, 11 are present in all 27 plots – seven broadleaved species: birch (*Betula pendula*, Betulaceae), hornbeam (*Carpinus betulus* L., Betulaceae), beech (*F. sylvatica* L., Fagaceae), ash (*Fraxinus excelsior* L., Oleaceae), aspen (*Populus* spp., Salicaceae), oak (*Quercus* spp., Fagaceae), and lime tree (*Tilia* spp., Malvaceae) and four coniferous species: larch (*Larix decidua* Mill., Pinaceae), Norway spruce (*P. abies* (L.) H.Karst., Pinaceae), Scots pine (*Pinus sylvestris* L., Pinaceae) and Douglas fir (*Pseudotsuga menziesii* (Mirb.) Franco, Pinaceae). These were used to answer two fundamental questions on the diversity and distribution of WIF in different tree species in the early phase of decomposition (3 years) (Purahong et al., 2018b). Using a high resolution culture-independent approach (next generation sequencing), we found that diverse WIF (1,254 OTUs) have already colonized deadwood at an early stage of decomposition, with a higher frequency of Ascomycota over Basidiomycota, and specific differences in their respective α-, β-and γ-diversities (Purahong et al., 2018b). Deadwood logs of coniferous species displayed higher WIF α- and γ-diversity than many of the analyzed broadleaved species, but *C. betulus* and *F. sylvatica* exhibited the highest β-diversity. We further found that the WIF community assembly of these 11 tree species was non-random, with a strong tree species preference, especially in broadleaved trees. In the same experiment, the γ-diversities (total detected WIF) determined by the culture-independent approach is ∼12 times higher than those obtained from fruiting body surveys (97 species) (Baber et al., 2016; Purahong et al., 2018b). The difference in the number of WIF detected using these two approaches is very large, however improving fruiting body surveys by increasing sampling collection and putting more effort into fungal identification to cover microfungi and Ascomycota can significantly reduce the differences in the total detected WIF to ∼2.5 times (∼500 species). The aim of fruiting body surveys according to Baber et al. (2016) is for a quick investigation and comparison of macrofungi (Basidiomycota: mainly Polyporales, Agaricales and Russulales and Ascomycota: mainly Xylariales and some Helotiales and Pezizales) in many plots and regions once a year during the peak fruiting season in autumn, so the richness results from this study may not cover all WIF species that produce fruiting bodies on deadwood. Although our previous study successfully demonstrated the significant effect of tree species on WIF diversity and revealed the existence of tree species preferences, the hierarchy and quantitative contributions of the factors shaping WIF diversity and community composition were not determined as part of this work (Purahong et al., 2018b).

In the follow up study presented here, we extend and deepen the study of WIF using our experimental set-up with 11 log species, and relate the molecular WIF community data to metadata on wood-physicochemical properties (density, moisture content, C, N, C:N and pH) and geographical locations of the logs. The aims were: (i) to determine and quantify the respective contributions of biotic and abiotic factors such as tree species identity, wood-physicochemical properties, forest management type and geographical locations on the WIF community composition and richness (α-diversity); (ii) to disentangle differences in the responses between the two main tree species groups (broadleaved and coniferous trees); and (iii) to unravel differences in WIF community composition and richness patterns between Ascomycota and Basidiomycota.

Based on the limited number of studies on factors affecting WIF community composition and diversity that used between one and three tree species (Rajala et al., 2012; Baldrian et al., 2016; Hoppe et al., 2016; Purahong et al., 2016a, 2017) and our previous results (Purahong et al., 2018b), we hypothesized that: (i) tree species, wood pH, wood water content, macronutrients and forest management type are the most important factors to explain the variance in overall WIF community composition and richness; (ii) different factors significantly correspond to overall WIF community composition and richness in broadleaved and coniferous trees; and (iii) different factors are responsible for variation in Ascomycota and Basidiomycota WIF community composition and richness.

## Materials and Methods

### WIF Community Data: Field Site, Experimental Design, Laboratories Procedures and Bioinformatics

We reanalysed the published WIF community data from 11 tree species (five families of broadleaved trees and one family of conifers) derived using next generation sequencing (Purahong et al., 2018b). This experiment was conducted in 27 experimental forest plots (1 ha each) distributed across Germany (North-East: Schorfheide-Chorin region (ca. 1300 km^2^; 53°01^′^N 13°77^′^E, 9 plots), Central Germany: Hainich-Dün region (ca. 1300 km^2^; 51°16^′^N 10°47^′^E, 9 plots) and South-Western: Schwäbische Alb region (ca. 422 km^2^; 48°44^′^N 9°39^′^E, 9 plots)) as part of the German Biodiversity Exploratories (Fischer et al., 2010). The experimental forest plots represent typical forest management types as well as the dominant tree species in each region (Fischer et al., 2010). All information pertaining to these forest plots has been described in detail elsewhere (Fischer et al., 2010; Hessenmöller et al., 2011). The freshly cut deadwood logs (winter 2008/2009) from each tree species [∼4 m long with a mean diameter of 31 ± 5.9 cm (mean ± SD)] were randomly placed in each forest plot beside each other with a distance of 1 m between logs in 2009 as part of the BELongDead project (Baber et al., 2016; Kahl et al., 2017). All logs were cut from the federal state of Thuringia (Germany) and then transported to each plot. The full experimental design consisted of 13 tree species, however only 11 tree species are present in all 27 plots (Purahong et al., 2018b). We thus, considered only these 11 species in this research (11 tree species; one log/species/site × 3 forest management types; Norway spruce age-class forest, European beech age-class forest and unmanaged beech forest × 3 locations; Schorfheide-Chorin region, Hainich-Dün region and Schwäbische Alb regions × 3 replicates = 297 logs in total).

Deadwood logs were sampled in September 2012 (3-years decomposition time), using a cordless drill (Makita BDF 451) equipped with a wood auger bit (diameter: 20 mm, length 450 mm) (Purahong et al., 2014a). The drill was operated slowly and introduced at an angle of ∼45° in relation to a vertical line perpendicular to the stem axis. We sampled deadwood at one position (50 cm from the tree base, two drills per log). Wood samples were subsampled for wood-physicochemical and molecular analyses. DNA was extracted from 100 mg of each homogenized wood sample using a ZR Soil Microbe DNA MiniPrep kit (Zymo Research, Irvine, CA, United States), according to the manufacturer’s instructions. For each wood sample we extracted DNA twice and the DNA extracts were pooled. The fungal internal transcribed spacer (ITS) rRNA region was amplified using the primer pair ITS1F (5^′^- CTTGGTCATTTAGAGGAAGTAA-3^′^), (Gardes and Bruns, 1993) and ITS4 (5^′^-TCCTCCGCTTATTGATATGC-3^′^), (White et al., 1990). We sequenced the samples from the ITS4 (reverse primer) using the PCR protocol described precisely elsewhere (Purahong et al., 2018b), then finally analyzed the ITS2 region (Purahong et al., 2016c). Products from three PCR replicates were then pooled in equimolar amounts and processed as according to Roche 454 protocols (454 Life Sciences, Branford, Connecticut, United States).

The raw demultiplexed reads were, first, quality trimmed using MOTHUR 1.33.3 (Schloss et al., 2009). High quality reads (containing one of the expected barcodes with a maximum of one mismatch; a minimum length of 300 nt; a minimum average quality Phred score of 20; containing homopolymers with a maximum length of 8 nt; and without ambiguous nucleotides) were shortened to their first 300 bases and normalized to the smallest read number per sample (3,011 reads). Potential chimeras were removed using UCHIME 4.2.40 (Edgar et al., 2011) as implemented in MOTHUR. Unique sequences were sorted according to decreasing abundance and were clustered into OTUs using CD-HIT-EST 4.5.4 (Fu et al., 2012) at a threshold of 97 % pairwise similarity. Fungal ITS OTU representative sequences were first classified against the dynamic version of the UNITE fungal ITS sequence database (Kõljalg et al., 2013) using the Bayesian classifier (Wang et al., 2007) as implemented in MOTHUR. The sequences with only fungi identified were further classified against the full version of the UNITE database in order to improve their taxonomic annotation. Rare OTUs (singletons to quadrupletons) could potentially have originated from sequencing errors (Kunin et al., 2010) and were therefore removed from the dataset. The raw sequence data sets are available in the European Nucleotide Archive under the study number PRJEB21052^1^. The taxonomic table for 1,254 WIF OTUs detected in this study is available in **Supplementary Table S1**.

### Wood-Physicochemical Analysis

For wood-physicochemical analysis, each wood sample was oven-dried and used to determine water content, wood density, C and N content and pH. The concentrations of C and N in wood samples were measured by total combustion using a Truspec elemental analyser (Leco, St. Joseph, MI, United States). Wood pH was measured for each sample in an aqueous extract (water) of a 0.5 g homogenised wood sample.

### Statistical Analysis

**FIGURE 1:**
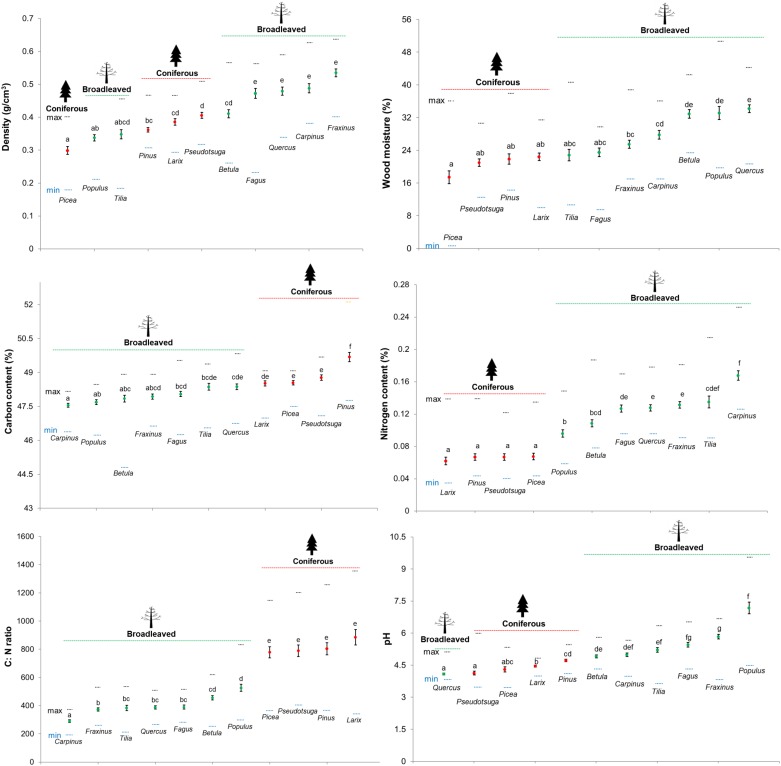
Wood physicochemical properties of deadwood logs decomposing for 3 years across 11 tree species (green = broadleaved tree species and red = coniferous tree species) in 27 forest plots (mean ± SE, *n* = 27). The maximum and minimum values are represented by the orange and blue dotted lines, respectively.

We performed a stepwise selection method with multiple regression analyses in SPSS (IBM SPSS Statistics 22, New York, NY, United States) to choose the best combination of variables that explained the differences in WIF richness. The alpha values selected to enter and remove independent variables were both set to 0.05. Normality of WIF richness datasets were tested using the Jarque-Bera JB test and the Chi-Square test using the function “normality” in PAST (Hammer et al., 2001). Multicollinearity was checked using a variance inflation factor (VIF): multicollinearity is considered to be present when the VIF is higher than 10. The details of the variables used in the analyses are shown in **Figure 1** and **Tables 1, 2**. All multivariate statistics were conducted on relative abundance data using the WIF dataset excluding 1–4 tons. Three dimensional-nonmetric multidimensional scaling (3D-NMDS) was conducted using the “vegan” package in R (Oksanen, 2013). The corresponding factors relating to fungal community composition were analyzed using the *envfit* function in “vegan,” with *P*-values being based on 999 permutations (Oksanen, 2013). The factors explaining WIF community composition were categorized into four groups: (i) tree species, (ii) wood-physicochemical properties: water content, density, C: N, C, N, pH, (iii) locations: Coordinates (N and E) and Exploratories (Schorfheide-Chorin region, Hainich-Dün region and Schwäbische Alb regions), and (iv) Forest management and analyzed to determine how much they and their interactions explained the variances in WIF community composition (variance partitioning analysis) using the *varpart* function in the vegan package in R. Detection of fungal OTUs with strong tree species preferences was performed using indicator species analysis (the ‘multipatt’ function, indicspecies package in R) (De Cáceres, 2013).

## Results

### Wood-Physicochemical Properties After 3 Years of Decomposition

The mean values of all measured wood-physicochemical properties in deadwood logs after 3 years of decomposition were clustered for broadleaved and coniferous tree species but most of these mean values (except C: N ratio and N content) of broadleaved and coniferous tree species were not significantly different (*P* > 0.05). Wood density was lowest in *P. abies* and highest in *F. sylvatica, Quercus* spp., *C. betulus* and *F. excelsior*. Wood moisture was also lowest in *P. abies* and highest in *Quercus* spp. Wood pH was lowest in *Quercus* spp. and *Pseudotsuga* and highest in *Populus* spp. N content was significantly lower and C: N ratio was significantly higher in all conifers than in broadleaved species. Some tree species had distinct wood-physicochemical property values, for example, *C. betulus* had the lowest C: N ratio (mean = 291.8; 186.9 – 375.3) and highest N content (mean = 0.17; 0.13 – 0.25), *P. sylvestris* had the highest C content, and *Populus* spp. had the highest pH (mean = 7.2; 4.5 – 9.5) (**Figure 1**). When considering the range of variation in measured wood-physicochemical properties, we found that the values for the different tree species partially overlapped and thus displayed a gradient that enabled us to separate the effects of wood-physicochemical properties from those of the tree species identity (**Figure 1**).

**Table 1:** Goodness-of-fit statistics (*R*^2^) for environmental and wood physicochemical factors fitted to the three dimensional non-metric multidimensional scaling (3D-NMDS) ordination of wood-inhabiting fungal community composition (^∗^*P* < 0.05, ^∗∗^*P* < 0.01, ^∗∗∗^*P* < 0.001).

Variable	All tree species			Broadleaved species			Coniferous species		
			
	All fungi	Ascomycota	Basidiomycota	All fungi	Ascomycota	Basidiomycota	All fungi	Ascomycota	Basidiomycota
**Tree species**									
Tree species identity	0.43^***^	0.42^***^	0.04^***^	0.39^***^	0.29^***^	0.24^***^	0.09^***^	0.07^**^	0.09^***^
**Space**									
Location (Region)	0.05^***^	0.01	0.01^***^	0.06^***^	0.01	0.04^***^	0.10^***^	0.02	0.10^***^
Coordinate_N	0.14^***^	0.01	0.05^***^	0.12^***^	0.03	0.10^***^	0.22^***^	0.02	0.20^***^
Coordinate_E	0.18^***^	0.01	0.05^***^	0.15^***^	0.04.	0.13^***^	0.26^***^	0.04	0.26^***^
**Wood physicochemical properties**									
Wood moisture content	0.20^***^	0.09^***^	0.09^***^	0.27^***^	0.29^***^	0.09^***^	0.28^***^	0.01	0.21^***^
Wood density	0.11^***^	0.14^***^	0.06^**^	0.11^***^	0.05^*^	0.15^***^	0.08^*^	0.06	0.08^*^
Carbon content (%)	0.14^***^	0.14^***^	0.18^***^	0.05^*^	0.03	0.09^**^	0.10^**^	0.02	0.11^**^
Nitrogen content (%)	0.34^***^	0.22^***^	0.30^***^	0.02	0.05^*^	0.03	0.04	0.07^*^	0.02
C: N ratio	0.36^***^	0.24^***^	0.31^***^	0.02	0.07^**^	0.05^*^	0.02	0.08^*^	0.01
Wood pH	0.31^***^	0.37^***^	0.23^***^	0.27^***^	0.27^***^	0.16^***^	0.21^***^	0.17^***^	0.20^***^
**Forest management**									
Forest management type	0.02	0.00	0.00	0.01	0.00	0.01	0.02	0.05	0.01


**Table 2:** Stepwise multiple regression analyses on wood-inhabiting fungal OTU richness as a function of eight independent variables (tree species, wood physicochemical properties and space).

Variable	*R*^2^ change	Standard coefficient β	*t*	*P*	Variance inflation factor (VIF)
**Total fungal richness in all tree species (multiple *R* = 0.44, *R*^2^ = 0.20 ), *F* = 17.82, *P* < 0.001)**					
Nitrogen content (%)	0.11	-0.22	-3.331	0.001	1.53
Location (exploratory)	0.04	-0.17	-3.242	0.001	1.04
Tree species identity	0.03	0.27	3.865	0.000	1.80
Wood density	0.02	0.16	2.63	0.009	1.33
**Ascomycota fungal richness in all tree species (multiple *R* = 0.39, *R*^2^ = 0.15 ), *F* = 12.90, *P* < 0.001)**					
Tree species identity	0.06	0.27	3.73	0.000	1.80
Location (exploratory)	0.05	-0.18	-3.18	0.002	1.04
Wood density	0.03	0.20	3.22	0.001	1.33
Nitrogen content (%)	0.01	-0.13	-2.00	0.047	1.53
**Basidiomycota fungal richness in all tree species (multiple *R* = 0.51, *R*^2^ = 0.26), *F* = 19.88, *P* < 0.001)**					
Nitrogen content (%)	0.16	-0.28	-4.40	0.000	1.53
Tree species identity	0.03	0.28	4.15	0.000	1.72
Wood moisture (%)	0.03	0.20	3.47	0.001	1.31
Wood pH	0.02	-0.15	-2.77	0.006	1.12
Management	0.02	-0.14	-2.73	0.007	1.02
**Total fungal richness in broadleaved species (multiple *R* = 0.47, *R*^2^ = 0.22 ), *F* = 12.67, *P* < 0.001)**					
Wood moisture (%)	0.12	0.26	3.76	0.000	1.14
Location (exploratory)	0.05	-0.18	-2.40	0.017	1.30
Wood pH	0.03	0.20	2.99	0.003	1.02
Carbon content (%)	0.02	0.14	1.97	0.050	1.17
**Ascomycota fungal richness in broadleaved species (multiple *R* = 0.41, *R*^2^ = 0.17), *F* = 12.68, *P* < 0.001)**					
Location (exploratory)	0.08	-0.25	-3.65	0.000	1.03
Wood pH	0.06	0.26	3.86	0.000	1.02
Wood density	0.03	0.18	2.58	0.011	1.04
**Basidiomycota fungal richness in broadleaved species (multiple *R* = 0.45, *R*^2^ = 0.20), *F* = 22.92, *P* < 0.001)**					
Wood moisture (%)	0.18	0.37	5.30	0.000	1.13
Location (exploratory)	0.02	-0.15	-2.20	0.029	1.13
**Total fungal richness in coniferous species (multiple *R* = 0.21, *R*^2^ = 0.05 ), *F* = 5.05, *P* = 0.027)**					
Nitrogen content (%)	0.05	-0.21	-2.25	0.027	1.00
**Ascomycota fungal richness in coniferous species (multiple *R* = 0.22, *R*^2^ = 0.05), *F* = 5.57, *P* = 0.020)**					
Nitrogen content (%)	0.05	-0.22	-2.36	0.020	1.00
**Basidiomycota fungal richness in coniferous species (multiple *R* = 0.29, *R*^2^ = 0.08), *F* = 5.57, *P* = 0.020)**					
Management	0.05	-0.20	-2.10	0.038	1.01
Carbon content (%)	0.04	-0.19	-2.02	0.046	1.01


The wood-physicochemical properties after 3 years of decomposition changed as compared with the initial wood-physicochemical properties (**Supplementary Figure S1**). Wood density in deadwood logs of all species except *Pseudotsuga* decreased by 11 – 24%. Total C slightly increased by 3 – 8%. For the dynamics of total N, three patterns were observed: N accumulation (increase, *Betula pendula, P. menziesii, P. sylvestris, Populus* spp.), N mineralization (decrease, *C. betulus, L. decidua*) and no accumulation/mineralization (stable, *Quercus* spp., *P. abies, Tilia* spp.*, F. sylvatica, F. excelsior*). N mineralization was clearly observed only in the two deadwood species with lowest initial C: N ratios [*C. betulus* (239), *L. decidua* (237.4)]. C: N ratios of *Betula pendula, P. menziesii, P. sylvestris* decreased greatly (15.2 – 50%) while those of *C. betulus, L. decidua* and *P. abies* increased markedly by 17.1–273%. The pH values of deadwood samples of most tree species changed substantially except *Pinus* and *Larix*.

### Determinants of WIF Community Composition: Interplay Among Tree Species, Wood Physicochemical Properties, and Locations

Goodness-of-fit statistics from 3D-NMDS ordination revealed that tree species identity was the most significant factor (*P* < 0.001) correlating with WIF community composition followed by wood-physicochemical properties. Among the wood-physicochemical properties, wood moisture and pH consistently yielded high correlations in both in broadleaved and coniferous species while N content and C: N ratio were no longer among the significant factors affecting the overall WIF community composition (**Table 1**). Environmental and wood physicochemical factors corresponding to fungal community composition in all locations and in each specific region are shown in **Supplementary Table S2**. Location (Exploratories and coordinates) significantly corresponded (*P* < 0.001) and explained the overall WIF community composition, however the goodness-of-fit statistics was low.

**FIGURE 2:**
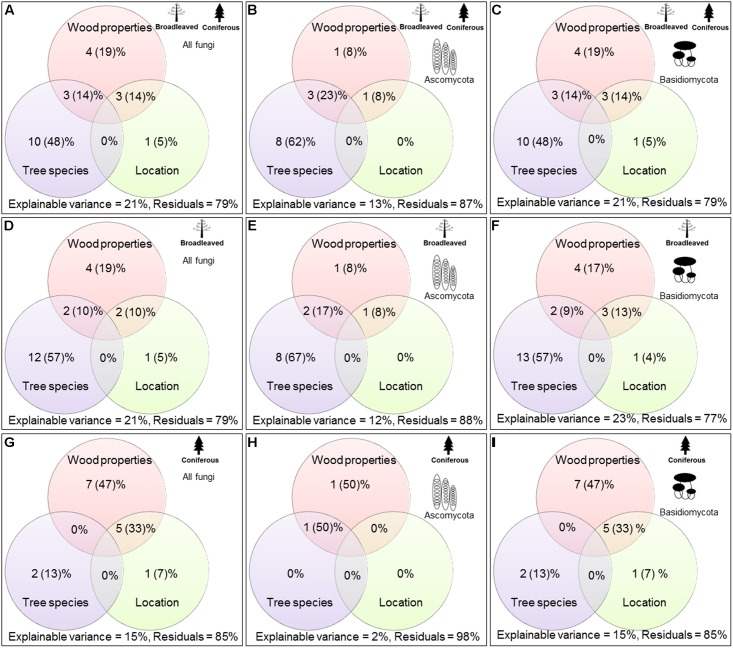
Variation partitioning analysis for evaluating the most important factors explaining the variations in wood-inhabiting fungal community composition (**A**,**D**,**G** = all fungi, **B**,**E**,**H** = Ascomycota, and **C**, **F**, **I** = Basidiomycota).

Variance partitioning analysis revealed that tree species identity was the most significant factor (*P* < 0.001), explaining 10% of the variance of WIF community composition, i.e., 48% of the variances could be explained by the measured factors. This was followed, in descending order of importance, by the effects of wood-physicochemical properties and the interactions between tree species and wood-physicochemical properties and between location and wood-physicochemical properties (**Figure 2**). Location factor alone explained a very low amount (1%) of the variance of the WIF community composition. Considering broadleaved and coniferous tree species separately, we found the results for broadleaved species to be similar to those found for all tree species (tree species > wood-physicochemical properties > interaction between tree species and wood-physicochemical properties and location and wood-physicochemical properties > location) (**Figure 2**). In contrast, in coniferous species, we found that wood-physicochemical properties explained more of the variance (7%, i.e., 47% of explainable variances) in WIF community composition than the other factors (interaction between location and wood-physicochemical properties > tree species > location) (**Figure 2**).

### Predictors of Total WIF OTU Richness

Nitrogen content explained the highest proportion of variances in WIF OTU richness. It was significantly negatively correlated with total WIF OTU richness when all tree species were considered, followed by location (increasingly negative from southwest to northeast), tree species identity (low in broadleaved and high in coniferous tree species) and wood density (positive) (**Table 2**). Deadwood N concentration was also identified as the only significant predictor of WIF OTU richness in coniferous species (negative correlation) (**Table 2**). Total fungal OTU richness in broadleaved trees was explained mainly by wood moisture content (positive), followed by location (increasingly negative from southwest to northeast), wood pH value (positive) and C concentration (positive) (**Table 2**).

Factors explaining WIF OTU richness and community composition in Ascomycota and Basidiomycota differed between broadleaved and coniferous trees.

When considering all tree species, Ascomycetous WIF OTU richness was mainly explained by tree species identity and location, while for Basidiomycota it was mainly explained by deadwood N content (**Table 2**). In broadleaved tree species, Ascomycetous WIF richness was mainly explained by location and wood pH, whereas in Basidiomycetous WIF was almost exclusively explained by wood moisture (**Table 2**). In coniferous tree species, only N content explained WIF Ascomycetous richness, while forest management and deadwood C content explained WIF Basidiomycota richness (**Table 2**).

Factors explaining the community composition of Ascomycota and Basidiomycota in broadleaved and coniferous tree species were also different (**Figure 2**). In broadleaved tree species, the Ascomycetous WIF community composition was mainly explained by tree species identity (8%), whereas for Basidiomycota, tree species identities and wood-physicochemical properties both explained the majority of variance in WIF community composition (4 – 13%). In coniferous tree species, the Ascomycetous WIF community composition was explained only to a small extent (1%) by wood-physicochemical properties and interactions between tree species identity and wood-physicochemical properties. For Basidiomycota, wood-physicochemical properties explained the majority of variance in WIF community composition (7%). Tree species identities and the interaction between wood-physicochemical properties and location did not explain any variation in Ascomycetous community composition, but these factors were significant for Basidiomycota (explaining 2 – 5%).

## Discussion

To the best of our knowledge, no other study has examined so many tree species to quantify the relative contributions of different factors to WIF community composition and richness determined using molecular techniques (Seibold et al., 2015). In addition, the analyses differentiated the effects within two groups of trees (broadleaved *vs*. coniferous trees) and two main fungal phyla (Ascomycota *vs*. Basidiomycota). The unequal number of broadleaved (7) *vs*. coniferous (4) tree species used in this experiment may have influenced our analysis when we considered all tree species, and the results must therefore be interpreted cautiously. The results for each group of trees considered separately may be more accurate and representative of the factors actually determining the WIF community composition and richness in the specific group of trees. Keeping possible biases inherent to molecular approaches in mind (Voříšková, 2013), our work provides important and novel insights into the factors that explain the distribution and richness patterns of WIF in temperate forests.

Although all deadwood logs used in the experiment came from the same geographic region in Germany (Thuringia forest, forest district Arnstadt), we have demonstrated that within each species, these logs exhibited greatly variable wood-physicochemical characteristics after 3 years of decomposition at different sites with different soils, geology (acid sands in the Schorfheide-Chorin region and limestone in the Hainich-Dün and Schwäbische Alb regions), forest structure and composition and previous management (Fischer et al., 2010; Hessenmöller et al., 2011). These differences corresponded significantly to the respective WIF communities. The initial physicochemical parameters of the deadwood logs used in this experiment have been published elsewhere (Kahl et al., 2017; **Supplementary Figure S1**). It is still debated whether changes in the WIF community alter physicochemical wood properties or whether these properties alter the WIF community (Purahong et al., 2016a). Recent next-generation sequencing studies suggest that the two are interdependent during the wood decomposition process (Hoppe et al., 2016; Mäkipää et al., 2017; Purahong et al., 2017). Initial physicochemical wood properties (pH, C, N and lignin content) and tree species identity select the WIF community (especially species originating from soil) that colonize deadwood (Mäkipää et al., 2017). Once these WIF have established and colonized the deadwood, they start decomposing the substrate but also compete with each other and with secondary infecting WIF (Purahong et al., 2017). By-products arise from the decomposition processes, and antagonisms (competition), which can influence the physicochemical wood properties, including C, N and pH, depending on WIF identities (Humar et al., 2001; Liers et al., 2011).

During decomposition, physicochemical wood properties change greatly as some groups of WIF (especially those WIF that form cords and rhizomorphs) can actively translocate nutrients (i.e., N) from other substrates such as litter and soil (Wells et al., 1998). In our experiment, we observed obvious pH changes in *Populus* spp. deadwood, as the values ranged from 4.5 to 9.5 after 3 years compared to 5.3 initially (Kahl et al., 2017). In this tree species, we found a significant correlation (ρ = 0.55, *P* = 0.003) between the relative abundances of *Pholiota populnea* OTU_0045 and deadwood pH. All wood-samples containing this fungus at a relative abundance higher than 20% had pH values ≥ 9, while in all samples with a pH lower than 5 this fungus was not detected (**Supplementary Table S3**). Effects of pH changes in wood resulting from activity of specific fungi have already been reported in an experiment investigating pH in wood samples exposed to different WIF under laboratory conditions (Humar et al., 2001).

In this work, we successfully quantified the relative contributions of different factors on WIF community composition at the end of the first 3 years of decomposition. Tree species and physicochemical wood properties (i.e., pH and N) are known to correlate significantly, thus it is very difficult, or perhaps impossible, to quantify the relative importance of each of these factors on WIF community composition (Baldrian et al., 2016). We applied variation partitioning analysis to evaluate the most important factors explaining variations in WIF community composition. This enabled us not only to compare the relative contribution of each factor but also their combined effects. Indeed, we noticed that the combined effect of tree species and physicochemical wood properties explained substantial proportions of WIF community composition (3% of all variances, which accounts for 14% of explainable variances).

We hypothesized that tree species identity, pH, macronutrients, geographical locations and forest management type were the most important factors explaining the majority of variances in the overall WIF community composition and richness (Rajala et al., 2012; Baldrian et al., 2016; Hoppe et al., 2016; Purahong et al., 2016a, 2017; Krah et al., 2018). This hypothesis was partly verified as we found that tree species identity (Krah et al., 2018) as well as some physicochemical wood properties, especially pH and macronutrients (N), significantly corresponded to and explained notable proportions of the total WIF community composition and richness when considering all tree species. However, the responses of WIF to factors varied greatly when the analyses were carried out separately for all species or only broadleaved or only coniferous trees, or when analyzing the two main fungal phyla, Ascomycota and Basidiomycota, separately.

N content in deadwood was one of the most important factors explaining WIF richness both when considering all tree species and all coniferous species (significant negative correlation), and this can be interpreted as reflecting an optimized resource partitioning in heterogeneous oligotrophic and N-depleted habitats. Baldrian et al. (2016) further suggest that N content is one of the most important drivers of fungal biomass content, WIF community composition and enzyme activity. Interestingly, the impact of geographical location and forest management on WIF community composition appeared to be small. This is also indicated by low importance of spatial coordinates on WIF community composition. In this study, geographical location reflected differences in climate and soil types and explains mainly the WIF richness (Fischer et al., 2010). The influence of location might have been stronger, if the deadwood logs had been sourced from each region instead of the one region. Furthermore, we only found a significant correlation between forest management and the overall WIF community composition in two (Schorfheide-Chorin and Schwäbische Alb) of the three regions and the Basidiomycota richness in coniferous species. This relative lack of importance of geographical location and forest management may reflect the efficiency of fungal dispersal via various mechanisms between the studied areas, where distances ranged from 0.315 km (within one region) to 626.9 km (the longest distance between plots in north-eastern and south-western Germany) (Purahong et al., 2016b). The forest management types within this experiment consist of unmanaged mixed forests as well as managed even-aged forests of European beech and conifers. Thus it can be considered that in each region WIF are homogeneously dispersed between plots of all management types. This observation suggests that maintaining different management types with different tree species compositions may be a good strategy to conserve WIF diversity in European temperate forests that display relatively low tree species diversity.

Determinants for the WIF community composition in logs of broadleaved and coniferous tree species differed. We found that tree species identity and physicochemical wood properties play important roles in shaping WIF community composition in broadleaved logs, while only physicochemical wood properties played a role in coniferous species. Wood pH is the only physicochemical wood parameter that consistently corresponds to WIF community composition (all, Ascomycetous and Basidiomycetous WIF) in logs of both broadleaved and coniferous tree species. The pH is among the most important factors determining mycelial growth, decomposition ability of plant materials and enzyme production by fungi (Kok et al., 1992). Effects of pH on fungal community composition have been found consistently across different habitats including soil, leaf litter and deadwood (Goldmann et al., 2015; Purahong et al., 2016c, 2017). Specific fungal communities and taxa can change wood pH to levels suitable for their growth and survival (Humar et al., 2001). For fungal pathogens, ammonia plays a key role in environmental alkalifying (raising the pH value), however it is still unclear which substances may have an alkalifying effect in deadwood (Vylkova, 2017). Ammonia can be also released to the environment from decomposition process of macrofungal fruitbodies (by microbial decomposition and from excretion of invertebrates consuming the fruitbodies) and causes an increase of soil pH (Ingelög and Nohrstedt, 1993). In deadwood, bacteria and archaea may also contribute to pH changes through the two versions of ammonification: (i) N fixation and (ii) anaerobic assimilatory and dissimilatory nitrite reduction to ammonium (Stein and Klotz, 2016). N fixing bacteria (Hoppe et al., 2014, 2015; Mäkipää et al., 2018; Probst et al., 2018) and known bacteria involve in dissimilatory nitrite reduction to ammonium (e.g., *Clostridium, Klebsiella*) (Spano et al., 1982; Johnston et al., 2016) are detected in conifer and broadleaved deadwood logs. Ammonium in deadwood can be then potentially converted to nitrate via nitrification process, hydrogen (H^+^) is released, which can decrease wood pH (Stein and Klotz, 2016). In fact, substantial amounts of ammonium and nitrate have been detected in our deadwood logs (Bantle et al., 2014). Wood moisture content and C content also correspond to WIF community composition for all and Basidiomycota WIF in both broadleaved and coniferous tree species. Water content is known to be an important factor for WIF in both broadleaved and coniferous tree species (Hoppe et al., 2016). Different tree species with different wood structure can affect free and bound water in deadwood logs differently. C is an important macronutrient essential for growth and reproduction of microorganisms (Purahong et al., 2016c). However, high carbon concentrations in wood are indicative of low concentrations of other nutrients and conifers tend to have higher wood C concentrations than broadleaved species (Thomas and Martin, 2012). Hence, higher concentrations of C in wood do not indicate a higher availability of C to microbes.

**FIGURE 3:**
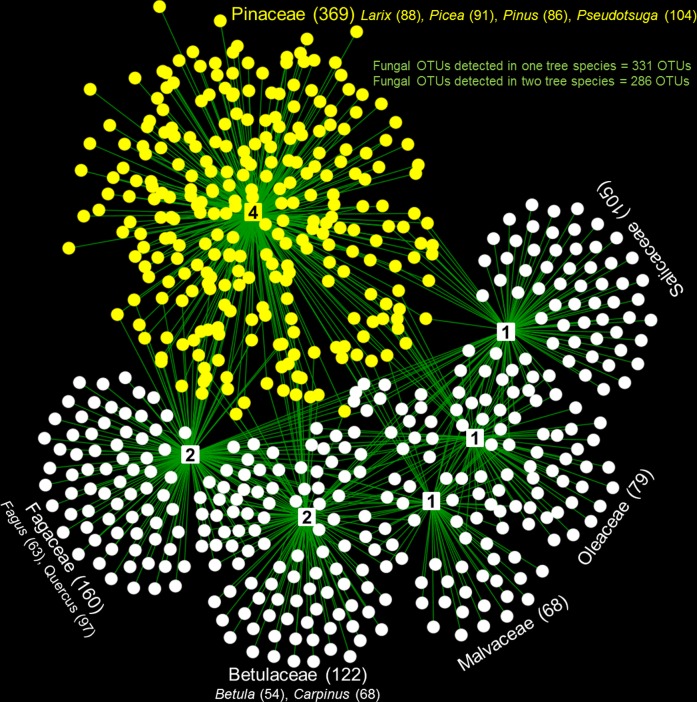
The overall architecture of tree family–fungal associations illustrates how wood-inhabiting fungal OTUs that show preferences for particular tree species (detected in no more than two tree species, in total 617 OTUs) were distributed within a web of wood-inhabiting fungi. Different node shapes represent different organismic groups: square nodes = plant families (number of tree species in each family is shown in the middle of each node) and circular nodes = fungi (yellow nodes = fungal OTUs associated with *Pinaceae* and white nodes = fungal OTUs associated only with broadleaved tree families; the numbers of fungal OTUs associated with each family and tree species are shown in brackets).

Tree species identity is a very broad factor that accounts for numerous biotic and abiotic attributes (i.e., tree species specific traits: micronutrient, macronutrients, celluloses, hemicellulose, lignin, etc.) not measured in either this or most other studies (Purahong et al., 2017), but it also partly includes phylogeny. The effect of tree phylogeny on WIF communities may arise from the coevolution between tree species and WIF (Purahong et al., 2018b), as already envisaged for symbiotic fungi (Brundrett, 2002). The broadleaved tree species analyzed in our experiment belong to different families, while the conifers analyzed belong all to the family Pinaceae (**Figure 3** and **Supplementary Figure S2**). While wood-traits associated with different tree families can vary greatly, the important physicochemical wood properties known to determine the WIF community composition such as wood structure, density, C and N content, C: N ratio and pH of distinct tree families (i.e., Oleaceae: *F. excelsior* and Fagaceae: *Quercus* spp.) can be very similar. We found the most different WIF community composition between *F. excelsior* and *Quercus* spp. despite similar physicochemical wood properties (Purahong et al., 2018b). This may be attributed to the tree phylogeny effects on the WIF community. The effect of tree phylogeny on WIF communities is also illustrated by the fact that WIF communities of phylogenetically close tree species are likely to be similar (less differences in *R*_ANOSIM_) (**Supplementary Figure S2**). The lower WIF heterogeneity in community composition in coniferous trees may simply reflect the phylogenetic closeness of the species examined. To confirm this assumption, the future experiment should include coniferous species from other families [i.e., Taxaceae (*Taxus*), Cupressaceae (*Juniperus*)]. However, an exception was found for the WIF community in *F. sylvatica* which was generally more similar to the WIF community of other tree species. The extremely high abundance of this tree species in the study areas may mean that its deadwood functions as a key reservoir of WIF biodiversity in forest ecosystems. Nevertheless, as the other broadleaved tree species harbor distinct WIF OTUs, they should be preserved to maintain the WIF diversity. The overall architecture of tree family–fungal associations illustrates how wood-inhabiting fungal OTUs that show preferences for particular tree species and families (detected in no more than two tree species, in total 617 OTUs) were distributed within a web of WIF (**Figure 3**). All WIF OTUs that exhibit potential preferences (402 OTUs) for particular tree species and tree species combinations according to the indicator species analysis are shown in **Supplementary Table S4**.

Apart from tree species effects and the distinction between broadleaved and coniferous trees, our study also provides evidence that factors explaining WIF diversity and community structure in Ascomycota and Basidiomycota are different. This may reflect the different requirements for growth and reproduction of these two fungal phyla and their distinct strategies to adapt to the different habitats represented by broadleaved and coniferous wood respectively (Küffer and Senn-Irlet, 2005). Ascomycota are believed to be more dependent on the availability of nutrients in the substrate than Basidiomycota. They are less capable of forming extended mycelial networks to translocate resources from other substrates (Purahong et al., 2016b). Furthermore, they also have a more limited ability compared with Basidiomycota to produce oxidative, lignin-degrading enzymes, especially Manganese peroxidase (MnP), so that they rely on the production of hydrolytic enzymes attacking more simple compounds in deadwood (Purahong et al., 2016b). Thus, the nutrient requirements for growth and enzyme production between Ascomycota and Basidiomycota may differ greatly.

## Conclusion

Overall, in this study we were able to determine some important factors and quantify their respective contributions in shaping WIF community composition and richness. We conclude that (i) tree species identity is the most significant factor, corresponding with the overall WIF community composition, (ii) wood pH is the only wood-physicochemical properties that consistently corresponded to WIF community composition, (iii) overall WIF richness patterns (∼20% of the total variance) are explained by wood N content, location, tree species identity and wood density and (iv) the importance of determinants of WIF community composition and richness appeared to depend greatly on tree species group (broadleaved *vs*. coniferous) and the fungal phyla (Ascomycota *vs*. Basidiomycota).

In this study, we could explain only ∼20 – 21% of WIF community composition and richness using the commonly measured factors such as tree species identity, physicochemical wood properties (C, N, C: N ratio, pH, moisture content) and location. The future experiment should include other groups of factors i.e., microclimate, elevation, plant community, etc., which may explain WIF community composition and richness (Purahong et al., 2017; Thorn et al., 2018). The results of this study relate only to the initial phase of wood decomposition (3 years), during which Ascomycota display higher OTU richness than Basidiomycota. We can speculate that during later stages of decomposition, during which availability of resources declines and Basidiomycota tend to dominate due to their stronger wood decaying potential, the contribution of factors that shape WIF community composition and species richness may change significantly.

## Ethics Statement

Field work permits were issued by the responsible environmental offices of Brandenburg, Baden-Württemberg and the Free State of Thuringia (according to §72 BbgNatSchG).

## Data Availability Statements

Datasets are in a publicly accessible repository: The datasets analyzed for this study can be found in the European Nucleotide Archive under the study number PRJEB21052 (http://www.ebi.ac.uk/ena/data/view/PRJEB21052).

## Author Contributions

FB, TK, DK, MH, JB, WW, E-DS, BH, TA, TW, and WP conceived and designed the experiments. WP, TA, TK, BH, KB, and PO performed the field experiments. WP and KJ performed the laboratory experiments. TW and GL performed the bioinformatics. WP analyzed the data. DK, FB, E-DS, and WW contributed reagents, materials, and analysis tools. WP wrote the manuscript. FB, TW, E-DS, WW, HK, MH, and JB commented and revised the manuscript. All authors have seen and approved the manuscript being submitted.

## Conflict of Interest Statement

The authors declare that the research was conducted in the absence of any commercial or financial relationships that could be construed as a potential conflict of interest.

## Footnotes

1 http://www.ebi.ac.uk/ena/data/view/PRJEB21052
