# Supplementary material for: Determinants of Deadwood-Inhabiting Fungal Communities in Temperate Forests: Molecular Evidence From a Large Scale Deadwood Decomposition Experiment
Source: Front Microbiol. 2018 Sep 20;9:2120. doi: 10.3389/fmicb.2018.02120 (PMC6158579; doi:10.3389/fmicb.2018.02120)
Supplement: Supplementary file 3 [file Presentation_1.pdf]

Supplementary material

Determinants of deadwood-inhabiting fungal communities in temperate forests: molecular evidence from a large scale deadwood decomposition experiment

Witoon Purahong[1]*, Tesfaye Wubet[1, 2]*ω, Guillaume Lentendu[1,3], Björn Hoppe[1,4], Katalee Jariyavidyanont[1], Tobias Arnstadt[5], Kristin Baber[6], Peter Otto[7], Harald Kellner[5], Martin Hofrichter[5], Jürgen Bauhus[8], Wolfgang W. Weisser[9], Dirk Krüger[1], Ernst-Detlef Schulze[10], Tiemo Kahl[8,11]¶, François Buscot[1, 2]¶.

[1]UFZ-Helmholtz Centre for Environmental Research, Department of Soil Ecology, Halle (Saale), Germany, [2]German Centre for Integrative Biodiversity Research (iDiv), Leipzig, Germany, [3]Department of Ecology, Technical University of Kaiserslautern, Kaiserslautern, Germany, [4]Julius Kühn-Institute, Institute for National and International Plant Health, Messeweg 11/12, D-38104 Braunschweig, Germany, [5]Department of Biology and Environmental Sciences, International Institute Zittau (IHI), Technische Universität Dresden, Zittau, Germany, [6]Institute of Biology, Department of Systematic Botany and Functional Biodiversity, University of Leipzig, Leipzig, Germany, [7]Institute of Biology, Department of Molecular Evolution and Plant Systematics, University of Leipzig, Leipzig, Germany, [8]Chair of Silviculture, Faculty of Environment and Natural Resources, University of Freiburg, , Freiburg i. Brsg., Germany, [9]Terrestrial Ecology Research Group, Department of Ecology and Ecosystem Management, School of Life Sciences Weihenstephan, Technische Universität München, Freising, Germany, [10]Max Planck Institute for Biogeochemistry, Jena, Germany,[11]Biosphere Reserve Vessertal-Thuringian Forest, Schmiedefeld am Rennsteig, Germany.

***Correspondence:**

Dr.Witoon Purahong (witoon.purahong@ufz.de);

Dr.Tesfaye Wubet (tesfaye.wubet@ufz.de).

¶Senior authors.

ωCurrent address: UFZ-Helmholtz Centre for Environmental Research, Department of Community Ecology, Halle (Saale), Germany

Table S2 Goodness-of-fit statistics (R^2) for environmental and wood physicochemical factors fitted to the three dimensional non-metric multidimensional scaling (3D-NMDS) ordination of fungal community composition in all locations and in each specific region (AEW = Schwäbische Alb, HEW = Hainich-Dün, SEW = Schorfheide-Chorin)

Factor	All locations		AEW		HEW		SEW	
	R^2	*P*	R^2	*P*	R^2	*P*	R^2	*P*
**All detected fungi**								
Geographical region	0.05	**0.001**	n.d	n.d	n.d	n.d	n.d	n.d
Tree species	0.42	**0.001**	0.31	**0.001**	0.52	**0.001**	0.51	**0.001**
Coordinate_N	0.15	**0.001**	0.02	0.612	0.01	0.762	0.04	0.286
Coordinate_E	0.19	**0.001**	0.06	0.123	0.10	**0.014**	0.00	0.928
Wood moisture content (%)	0.20	**0.001**	0.16	**0.001**	0.16	**0.001**	0.18	**0.002**
Density (g/cm3)	0.11	**0.001**	0.15	**0.003**	0.16	**0.003**	0.12	**0.007**
Management	0.02	0.12	0.13	**0.006**	0.03	0.464	0.08	**0.048**
Carbon content (%)	0.14	**0.001**	0.20	**0.001**	0.10	**0.017**	0.20	**0.001**
Nitrogen content (%)	0.34	**0.001**	0.24	**0.001**	0.47	**0.001**	0.40	**0.001**
C: N ratio	0.35	**0.001**	0.35	**0.001**	0.44	**0.001**	0.38	**0.001**
Wood pH	0.28	**0.001**	0.36	**0.001**	0.32	**0.001**	0.35	**0.001**
**Ascomycota**								
Geographical region	0.00	0.782	n.d	n.d	n.d	n.d	n.d	n.d
Tree species	0.41	**0.001**	0.51	**0.001**	0.61	**0.001**	0.47	**0.001**
Coordinate_N	0.01	0.591	0.03	0.378	0.01	0.840	0.02	0.698
Coordinate_E	0.01	0.551	0.05	0.205	0.04	0.281	0.01	0.685
Wood moisture content (%)	0.08	**0.001**	0.04	0.276	0.17	**0.002**	0.03	0.323
Density (g/cm3)	0.16	**0.001**	0.25	**0.001**	0.21	**0.001**	0.03	0.36
Management	0.01	0.654	0.04	0.222	0.01	0.804	0.06	0.138
Carbon content (%)	0.13	**0.001**	0.25	**0.001**	0.13	**0.003**	0.16	**0.004**
Nitrogen content (%)	0.20	**0.001**	0.21	**0.001**	0.39	**0.001**	0.18	**0.001**
C: N ratio	0.23	**0.001**	0.30	**0.001**	0.41	**0.001**	0.16	**0.003**
Wood pH	0.37	**0.001**	0.45	**0.001**	0.53	**0.001**	0.29	**0.001**
**Basidiomycota**								
Geographical region	0.01	**0.001**	n.d	n.d	n.d	n.d	n.d	n.d
Tree species	0.05	**0.001**	0.20	**0.014**	0.42	**0.001**	0.11	0.307
Coordinate_N	0.09	**0.001**	0.04	0.308	0.01	0.743	0.02	0.580
Coordinate_E	0.10	**0.001**	0.03	0.446	0.03	0.467	0.01	0.800
Wood moisture content (%)	0.08	**0.001**	0.10	**0.017**	0.17	**0.001**	0.18	**0.001**
Density (g/cm3)	0.18	**0.001**	0.10	**0.017**	0.17	**0.002**	0.22	**0.001**
Management	0.02	0.100	0.07	**0.046**	0.01	0.775	0.03	0.345
Carbon content (%)	0.11	**0.001**	0.19	**0.009**	0.04	0.270	0.31	**0.001**
Nitrogen content (%)	0.24	**0.001**	0.07	**0.066**	0.43	**0.001**	0.28	**0.001**
C: N ratio	0.25	**0.001**	0.16	**0.003**	0.35	**0.001**	0.30	**0.001**
Wood pH	0.11	**0.001**	0.28	**0.001**	0.15	**0.004**	0.02	0.449

Table S3. Relative abundances of *Pholiota populnea* OTU_0045 (%) and pH values in 27 *Populus* spp. wood samples (Spearman rank correlation ρ = 0.55, *P* = 0.003).

Tree species	pH	Relative abundance of *Pholiota populnea* OTU_0045 (%)
Populus spp.	4.49	0.00
Populus spp.	4.66	0.00
Populus spp.	4.82	0.00
Populus spp.	6.17	0.00
Populus spp.	7.74	0.00
Populus spp.	7.83	0.00
Populus spp.	5.45	0.03
Populus spp.	8.55	0.21
Populus spp.	8.23	0.24
Populus spp.	7.07	0.60
Populus spp.	6.6	0.64
Populus spp.	6.28	0.75
Populus spp.	7.99	0.84
Populus spp.	7.23	0.93
Populus spp.	6.39	1.17
Populus spp.	7.34	1.36
Populus spp.	6.44	1.80
Populus spp.	6.3	1.90
Populus spp.	8.75	2.51
Populus spp.	7.26	2.82
Populus spp.	7.41	3.74
Populus spp.	6.93	3.87
Populus spp.	9.4	5.76
Populus spp.	7.03	5.96
Populus spp.	**8.96**	**21.15**
Populus spp.	**9.54**	**27.76**
Populus spp.	**9.15**	**32.58**

Figure S1. Physicochemical factors of wood samples collected in 2009 (initial values, reproduce from Kahl et al., 2017) and in 2012 (this study). Red and green highlights indicate >10% decrease and increase of the values from year 2012 as compared to 2010.

Tree genus	Density g/cm3 (2009)	Density g/cm3 (2012)	Difference (%)	C%(2009)	C%(2012)	Difference (%)	N% (2009)	N% (2012)	Difference (%)	C:N ratio (2009)	C:N ratio (2012)	Difference (%)	pH (2009)	pH (2012)	Difference (%)
Betula	0.52	0.41	-20.18	45.60	47.85	4.94	0.06	0.11	80.87	760.00	456.98	-39.87	5.60	4.92	-12.13
Fagus	0.60	0.47	-21.71	45.80	48.05	4.92	0.12	0.13	5.68	381.67	391.12	2.48	4.40	5.46	24.18
Pseudotsuga	0.41	0.41	0.00	46.60	48.78	4.68	0.05	0.07	33.70	932.00	790.56	-15.18	6.00	4.14	-31.00
Quercus	0.59	0.48	-18.04	46.10	48.38	4.94	0.13	0.13	-1.82	354.62	388.02	9.42	5.00	4.09	-18.14
Fraxinus	0.61	0.54	-11.96	46.00	47.94	4.23	0.12	0.13	9.54	383.33	374.00	-2.44	4.20	5.84	39.11
Picea	0.36	0.30	-17.32	46.60	48.56	4.20	0.07	0.07	-3.49	665.71	779.50	17.09	5.00	4.32	-13.70
Carpinus	0.64	0.49	-23.94	45.40	47.56	4.76	0.19	0.17	-11.72	238.95	291.81	22.12	5.80	5.00	-13.81
Pinus	0.41	0.36	-11.92	48.30	49.70	2.89	0.03	0.07	122.47	1610.00	804.91	-50.01	4.70	4.73	0.71
Larix	0.43	0.39	-11.04	45.10	48.53	7.60	0.19	0.06	-67.39	237.37	885.70	273.13	4.60	4.47	-2.91
Tilia	0.45	0.35	-21.65	46.80	48.37	3.36	0.13	0.13	3.79	360.00	384.71	6.86	5.80	5.22	-9.94
Populus	0.44	0.34	-22.83	45.70	47.70	4.37	0.08	0.10	19.95	571.25	527.05	-7.74	5.30	7.19	35.58

Figure S2. Phylogenetic relationships of all tree species used in this study and the *R* ANOSIM showing the pair-wise difference between tree species (R = 0 – 0.24 no separation to barely separated, $R \geq 0.25 – 0.75$ separation with different degrees of overlap, $R > 0.75 – 1$ well separated to complete separation; significant P values ($P < 0.05$) are based on 9999 permutations and Bonferroni-corrections in all cases).

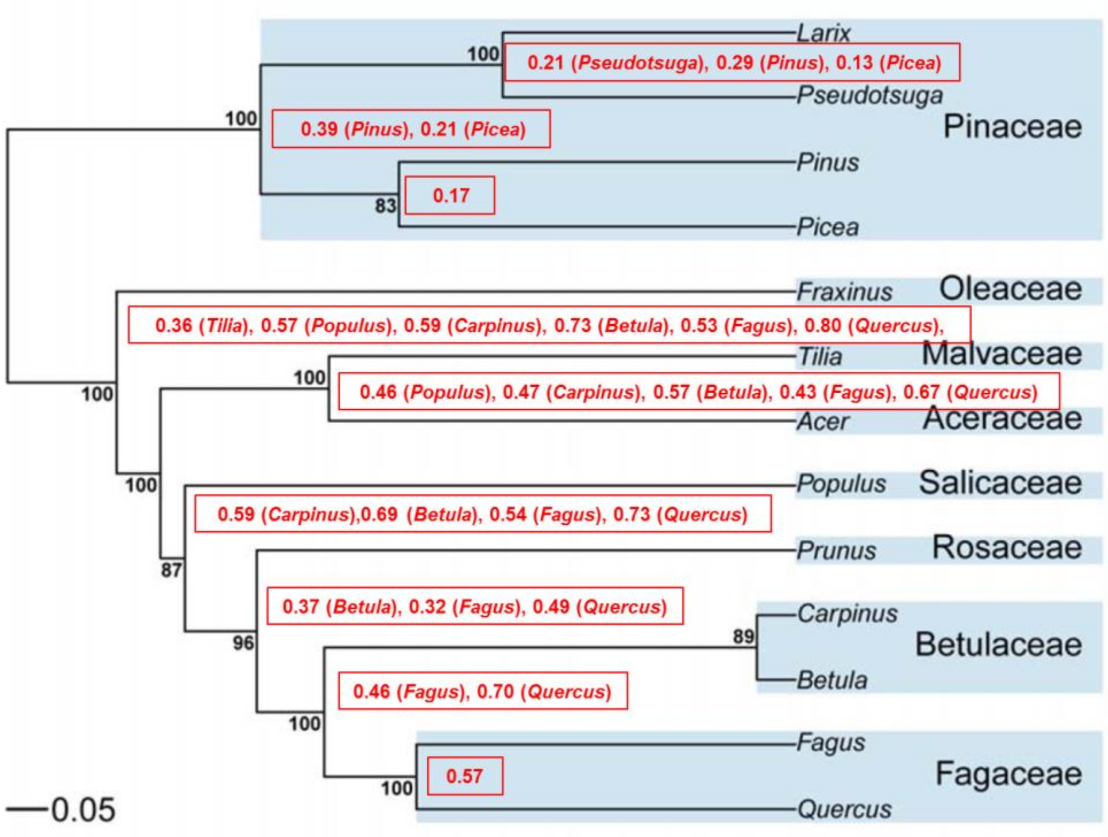

References

Kahl, T., Arnstadt, T., Baber, K., Bässler, C., Bauhus, J., Borken, W., et al. (2017). Wood decay rates of 13 temperate tree species in relation to wood properties, enzyme activities and organismic diversities. *For. Ecol. Manag.* 391, 86–95. doi:10.1016/j.foreco.2017.02.012.
